# Odonto-onycho-dermal dysplasia in a patient homozygous for a *WNT10A* nonsense mutation and mild manifestations of ectodermal dysplasia in carriers of the mutation

**DOI:** 10.1186/s12895-016-0040-7

**Published:** 2016-03-10

**Authors:** Anne Bruun Krøigård, Ole Clemmensen, Hans Gjørup, Jens Michael Hertz, Anette Bygum

**Affiliations:** Department of Clinical Genetics, Odense University Hospital, Sdr. Boulevard 29, DK-5000 Odense, Denmark; Department of Clinical Pathology, Odense University Hospital, Odense, Denmark; Department of Maxillofacial Surgery, Center for Oral Health in Rare Diseases, Aarhus University Hospital, Aarhus, Denmark; Department of Clinical Research, University of Southern Denmark, Odense, Denmark; Department of Dermatology and Allergy Centre, Odense University Hospital, Odense, Denmark

**Keywords:** Odonto-onycho-dermal dysplasia, OODD, Ectodermal dysplasia, *WNT10A* gene, Oligodontia

## Abstract

**Background:**

Odonto-onycho-dermal dysplasia (OODD) is a rare form of ectodermal dysplasia characterized by severe oligodontia, onychodysplasia, palmoplantar hyperkeratosis, dry skin, hypotrichosis, and hyperhidrosis of the palms and soles. The ectodermal dysplasias resulting from biallelic mutations in the *WNT10A* gene result in highly variable phenotypes, ranging from isolated tooth agenesis to OODD and Schöpf-Schulz-Passarge syndrome (SSPS).

**Case presentation:**

We identified a female patient, with consanguineous parents, who was clinically diagnosed with OODD. Genetic testing showed that she was homozygous for a previously reported pathogenic mutation in the *WNT10A* gene, c.321C > A, p.Cys107*. The skin and nail abnormalities were for many years interpreted as psoriasis and treated accordingly. A thorough clinical examination revealed hypotrichosis and hyperhidrosis of the soles and dental examination revealed agenesis of permanent teeth except the two maxillary central incisors. Skin biopsies from the hyperkeratotic palms and soles showed the characteristic changes of eccrine syringofibroadenomatosis, which has been described in patients with ectodermal dysplasias. Together with a family history of tooth anomalies, this lead to the clinical suspicion of a hereditary ectodermal dysplasia.

**Conclusion:**

This case illustrates the challenges of diagnosing ectodermal dysplasia like OODD and highlights the relevance of interdisciplinary cooperation in the diagnosis of rare conditions.

## Background

Odonto-onycho-dermal dysplasia (OODD) (OMIM# 257980) [1], is a rare autosomal recessive inherited form of ectodermal dysplasia, first reported in 1983 [2] and later further delineated by others [3–7]. The phenotypic appearance includes severe oligodontia, onychodysplasia, palmoplantar hyperkeratosis, dry skin, hypotrichosis, and hyperhidrosis of the palms and soles. A smooth tongue with marked reduction of fungiform and filiform papillae has also been reported [4].

OODD is caused by biallelic mutations in the *WNT10A* gene. *WNT10A* is a member of the *WNT* gene family, and is found to activate the canonical Wnt pathway, a signal transduction pathway essential for development of tissues of ectodermal origin [8]. Mutations in the *WNT10A* gene cause a broad spectrum of ectodermal dysplasias, ranging from mild signs of ectodermal dysplasia such as hypodontia to syndromes like OODD and Schöpf-Schulz-Passarge syndrome (SSPS). The clinical phenotype of SSPS includes numerous cysts along the eyelid margins in addition to palmoplantar keratoderma, hair, nail and teeth abnormalities [9]. *Wnt10A* has been recognized as a key regulator of odontogenesis and is found to be involved in regulation of odontoblast differentiation [10]. This is in line with recent evidence that *WNT10A* mutations are a common cause of non-syndromic tooth agenesis [11, 12].

According to the Human Gene Mutation Database, 66 different mutations in the *WNT10A* gene have been reported, of which 58 are missense and nonsense mutations [13]. Until now only around 30 patients with OODD have been reported in the literature [2, 4–7, 14–17]. However, the exact figure is difficult to estimate due to a lack of clear phenotypic distinction between the ectodermal dysplasias. Our report on a case of OODD emphasizes the challenges of diagnosing rare conditions like OODD. The condition is probably underreported, since a causal relation between skin, nail, hair and teeth abnormalities is needed in order to suspect the diagnosis.

## Case presentation

**Fig. 1:**
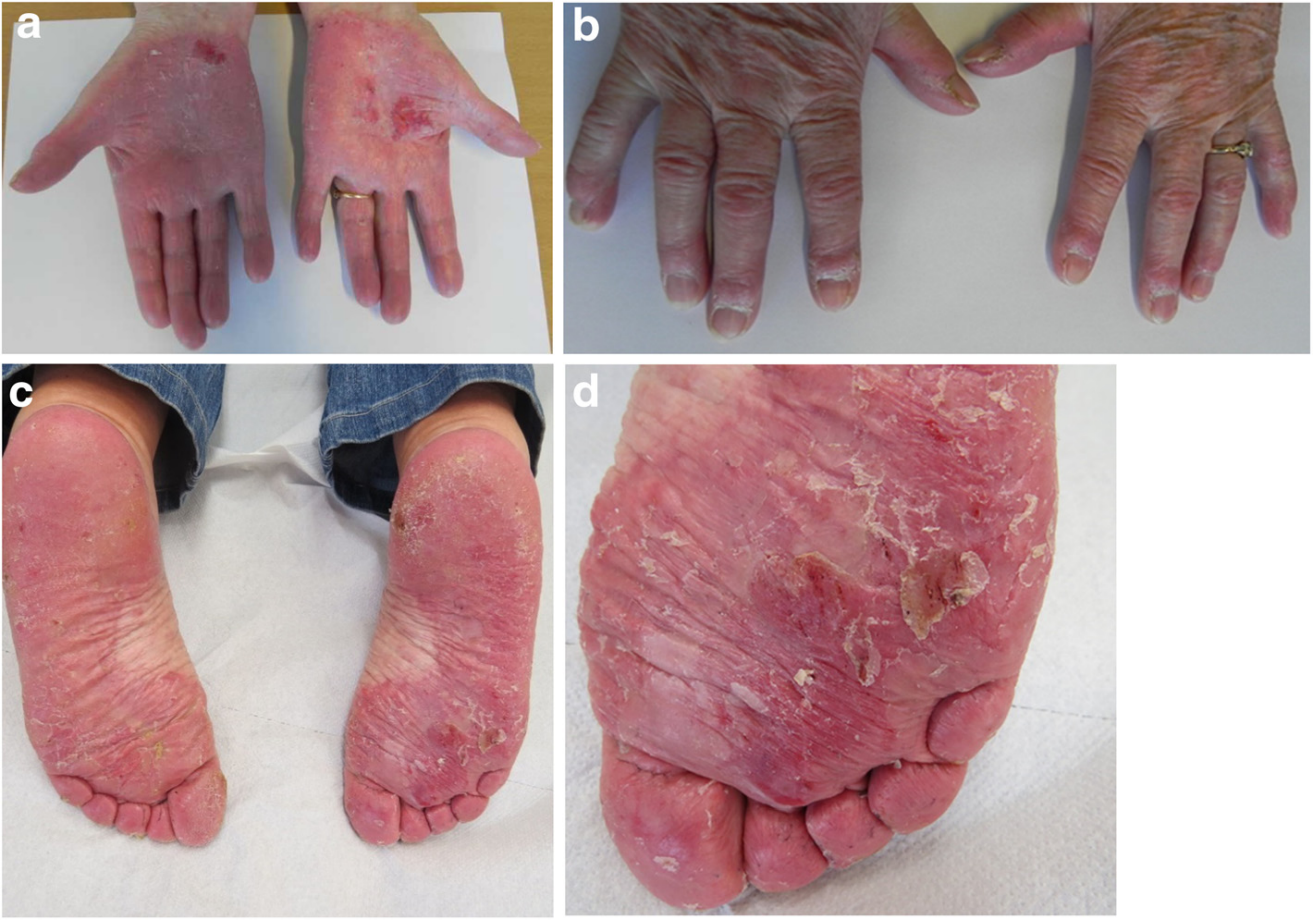
*Upper panel*: Hyperkeratosis of the palms with erythema, scaling, erosions and fissuring. Dystrophic finger nails with spooning. *Lower panel*: Hyperkeratosis of the soles with erythema, scaling, erosions and fissuring

**Fig. 2:**
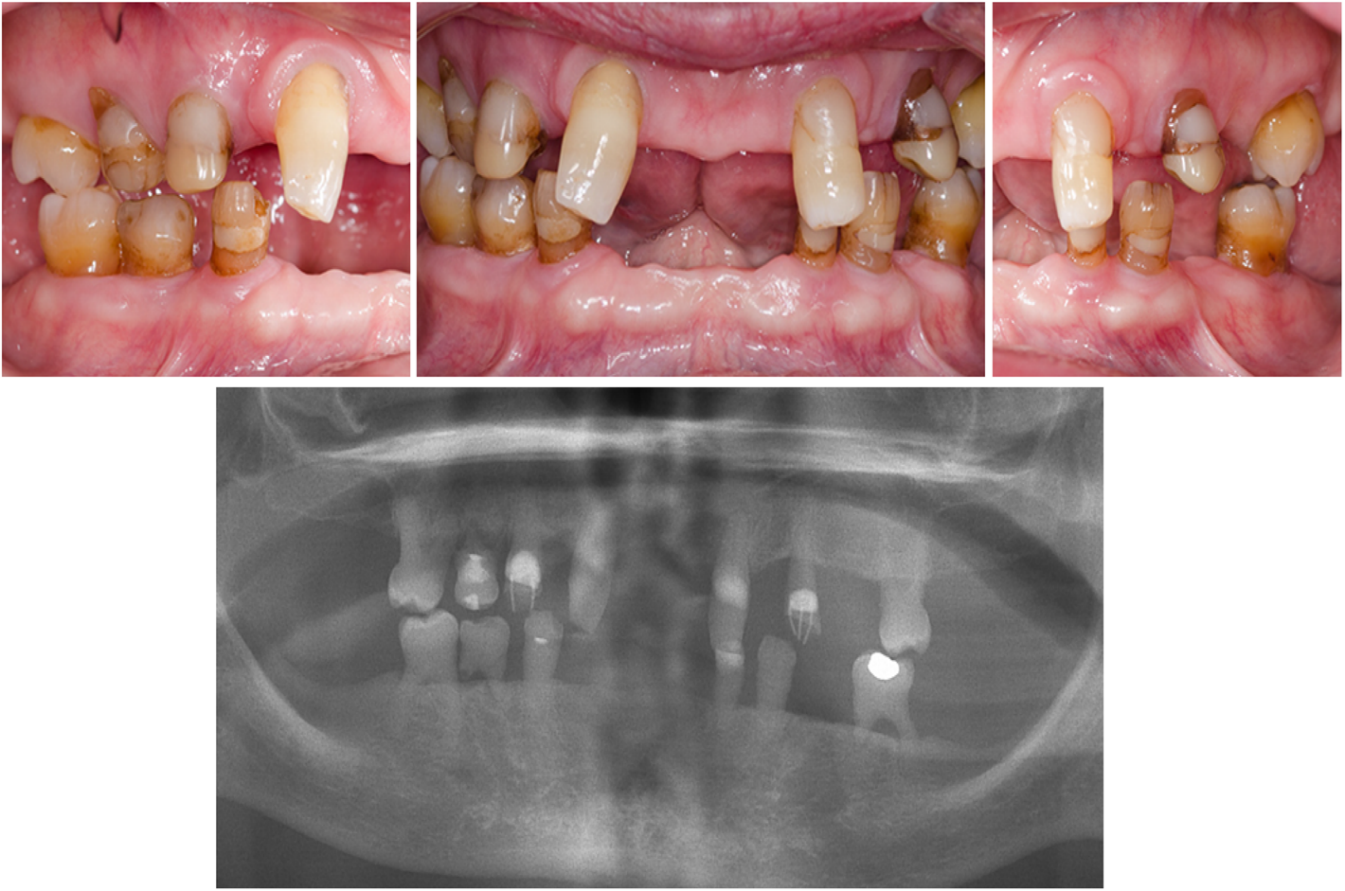
*Upper panel*: Intraoral photos of natural teeth in occlusion. *Lower panel*: Panoramic radiograph of the dentition. Preserved decidious dentition (11 decidious teeth), two screwdriver-shaped permanent incisors, and a large diastema mediale. Agenesis of all permanent teeth except two

The female patient, now aged 75, had since early childhood been affected by skin, tooth and nail abnormalities, but was only recently diagnosed with OODD. From early life she developed a blistering erythema and thick scaling on palms and soles (Fig. 1). She had dry skin, slightly dystrophic and brittle finger and toe nails with spooning. She reported abnormal sweating on the feet, which aggravated blistering of the skin. Since adolescence she reported hypotrichosis and in adulthood developed a bald patch on the scalp. She showed erythematous plaques on both cheeks and the nose and had two facial basal cell carcinomas surgically removed. At the time of our examinations, she had many of her deciduous teeth (six molars, four canines, and one mandibular incisor). Only two permanent teeth were present (maxillary incisors), and they were screwdriver-shaped. According to the patient’s memory, only one tooth had been extracted. Through adulthood deciduous teeth were maintained and supplemented by removable dentures (Fig. 2). At present, the prognosis of three of the molars is very poor.

Until the age of 75, the causal relation between these symptoms was not recognized and the skin and nail manifestations were interpreted as psoriasis and treated accordingly. Skin biopsies from the palms and soles showed an acrosyringial proliferation of anastomosing, epithelial cords and strands extending from the epidermis into a fibrovascular stroma. The epithelial cells were small and uniform, and disclosed a ductal differentiation focally. These findings correspond to those described for eccrine syringofibroadenomatosis (ESFA).

## Genetics

Genomic DNA purified from a blood sample from the patient was analyzed at All Wales Molecular Genetics Laboratory, Institute of Medical Genetics, UK using bidirectional Sanger sequencing of the *WNT10A* gene. The patient was homozygous for a nonsense mutation in the *WNT10A* gene, c.321C > A, p.Cys107*.

## Family history

**Fig. 3:**
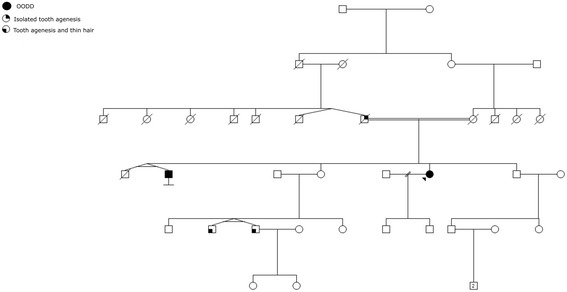
Family pedigree

The family history revealed consanguinity as the parents were first cousins, in accordance with autosomal recessive inheritance (Fig. 3). The patient’s brother had a phenotypic presentation compatible with severe OODD, but was never clinically diagnosed with the syndrome and genetic testing was never performed.

The patient’s father had small, conical teeth, most likely deciduous teeth, and had artificial dentition in adult life. He had no other symptoms and thus, presented with isolated tooth agenesis. Genetic testing was never performed. The patient’s mother, also an obligate carrier of the mutation, was completely asymptomatic. It is not known whether the patient’s grandparents had any symptoms. The patient’s nephews also presented with tooth agenesis and thin hair. Genetic testing was not performed, but it is speculated that they were heterozygous for the *WNT10A* mutation.

## Discussion

We present a female case of OODD homozygous for c.321C > A, p.Cys107* in *WNT10A*, which is a previously reported pathogenic mutation. In another family affected by ectodermal dysplasia, a male patient homozygous for the exact same mutation had clinical features of SSPS, whereas individuals who were compound heterozygous for p.Cys107*/p.Phe228Ile displayed a phenotype akin to OODD [18]. This illustrates lack of a clear genotype-phenotype correlation in these conditions.

The c.321C > A, p.Cys107* reported here, is previously reported in nine patients clinically diagnosed with OODD [14, 16, 18, 19], one patient with oligodontia and maxillary bone hypoplasia [20], 17 patients with isolated tooth agenesis [11, 21] and in nine patients with SSPS [9, 16, 18, 19]. The mutation is seen in both homozygous and compound heterozygous state and homozygous individuals are generally more severely affected than compound heterozygous individuals. This is not surprising, as c.321C > A, p.Cys107* is predicted to result in premature termination of the sequence which may lead to nonsense-mediated decay of the mRNA, whereas missense mutations most likely affect protein function to a lesser extent. As the clinical presentation of patients with the same genotype ranges from mild symptoms of ectodermal dysplasia to more severe syndromic manifestations, it is recently concluded that OODD and SSPS should be considered as variable expression of the same *WNT10A* genotype [19].

Our patient presented with two basal cell carcinomas prior to being diagnosed with OODD. Basal cell carcinomas have been described in other patients with OODD and an increased skin tumor risk in these patients has been suggested [16]. However, the presence may be coincidental as basal cell carcinoma occurs in 14 % of elderly people [22].

The fact that the patient’s father and nephews presented with tooth agenesis and tooth agenesis and thin hair, respectively, is in line with observations that heterozygous carriers may display minor disease-associated symptoms. It has been reported that about 50 % of individuals heterozygous for mutations in *WNT10A* display a phenotypic manifestation of abnormal Wnt signaling, such as abnormal shape or agenesis of one or several permanent teeth, nail dystrophy, dry skin, palmoplantar hyperkeratosis, sparse scalp hair, sparse eyelashes or sparse eyebrows [16]. In isolated oligodontia, a great proportion (up to 54 %) of the cases are associated by *WNT10A* mutations [23, 24]. In a population-based study on patients with oligodontia, *WNT10A* mutations accounted for 25 % of the cases, and it was shown that all biallelic *WNT10A* mutations were associated with absence of maxillary and mandibular molars as well as mandibular central incisors but presence of maxillary central incisors [23]. This is in accordance with the dental phenotype of the present OODD-case. A tendency of sex-biased manifestation pattern was reported in heterozygous individuals with a slightly higher proportion of tooth anomalies in males compared to females, which may implicate gender-specific differences in *WNT10A* expression [16].

Eccrine syringofibroadenoma is a rare, benign adnexal tumor histopathologically characterized by proliferation of anastomosing cords and strands of basaloid acrosyringeal cells, ductal differentiation and a mucinous fibrovascular stroma. Identical histopathological changes are seen in the diffuse variant (ESFA), which characteristically is located at the acral skin of palms and soles [25]. ESFA has been described in association with ectodermal dysplasia, unspecified as well as specified (SSPS), and in a review of 44 patients with eccrine syringofibroadenoma, ten patients among 17 with multiple or diffuse ESFA fulfilled the clinical criteria of SSPS [26]. This suggested that our patient should be examined for ectodermal dysplasia.

## Conclusion

We present a female patient diagnosed with OODD, which is a rare autosomal recessive inherited form of ectodermal dysplasia. The patient’s skin and nail manifestations were for many years interpreted as psoriasis and treated accordingly. This highlights the importance of including the full clinical picture comprising dental examination, histopathological examination and family history in order to establish the diagnosis of rare conditions like SSPS and OODD.

## Consent

Written informed consent was obtained from the patient for publication of this Case report and any accompanying images. A copy of the written consent is available for review by the Editor of this journal.

## Abbreviations

OODD: odonto-onycho-dermal dysplasia
SSPS: Schöpf-Schulz-Passarge syndrome
ESFA: eccrine syringofibroadenomatosis
